# 2D and 3D CT Diagnosis of Traumatic Incudostapedial Joint Separation

**DOI:** 10.5334/jbr-btr.831

**Published:** 2015-09-15

**Authors:** B. Coulier, Ch. Lamarque, St. Dejardin

**Affiliations:** 1 Department of Diagnostic Radiology, Clinique St Luc, Bouge (Namur), Belgium; 2 Department of Otorhinolaryngology, Clinique St Luc, Bouge (Namur), Belgium

A 55-year-old woman was referred to our department of Diagnostic Radiology for persistent conductive hearing loss on the left side (30 to 40 decibels in all frequencies) accompanied by dizziness in the waning of a serious head injury. Three months before the patient had heavily fell on the head in a stairwell with the result of diffuse cerebral (intraparenchymal hematoma) and extracerebral (subdural hematoma and subarachnoid hemorrhage) lesions and that mostly at the contralateral right cerebral hemisphere. The patient remained in coma for three weeks. High-definition multidetector CT showed a left middle ear which was actually completely free of residual fluid or blood allowing a perfect 2D and 3D analysis of the petrous bone and of the ossicular chain. A thin fracture was crossing the squamous part of the temporal bone to extend through the wall of the external auditory. It was prolongated by a longitudinal fracture of the temporal petrous through the middle ear (black arrow on Fig. A). 2D multiplanar reconstructions followed by selective 3D volume rendering of the ossicles perfectly identified an incudostapedial joint separation (white arrow on Fig. B).

**Figures A–B:**
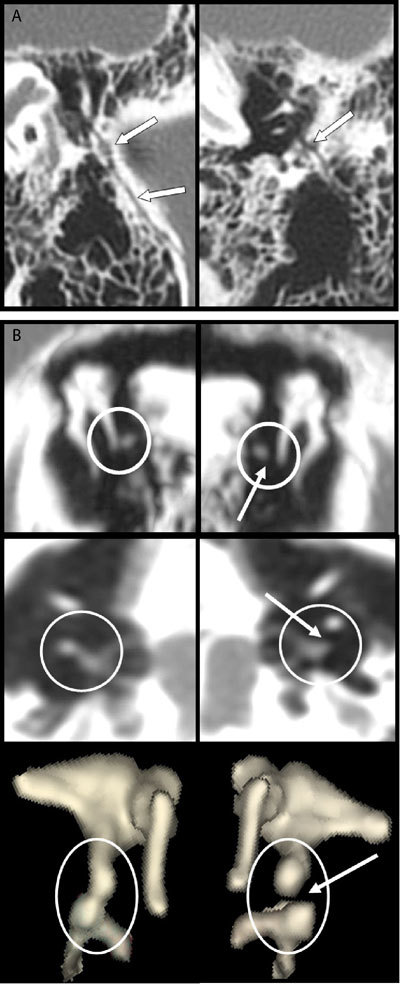


## Comment

Trauma of the middle ear usually manifests as conductive hearing loss that can be due to laceration of the tympan, hematotympanum or ossicular damage. If the hearing deficit persists after resolution of the hemotympanum or healing of the tympanic membrane, then ossicular dislocation or fracture is obvious.

Fractures of the temporal bone can be categorized into three types (longitudinal, transverse, or mixed) on the basis of their orientation relative to the long axis of the petrous temporal bone. Longitudinal fractures are the most common. They cross the middle ear and are often associated with ossicular dislocation or fracture. High resolution multidetector CT is the method of choice for evaluation of ossicular trauma.

There are five main types of dislocation: incudomalleolar and incudostapedial joint separation, dislocation of the incus or of the malleolarincudal complex and finally stapediovestibular disclocation. Stapedovestibular disclocation and fractures of the ossicula are rare.

Among these various types incudostapedial dislocation or disarticulation is the most common posttraumatic injury of the ossicular chain that and is most often associated with longitudinal fractures. The two main reasons are 1) the nature of tenuous suspension of the incus (the heavier ossicule that has no muscular anchor and the weakest attachments of the chain) between the malleus and stapes that are both firmly anchored and 2) the fact that the uncudostapedial is only a fragile enarthrosis which is usually the first injured.

Another mechanism has been postulated and could result from the simultaneous traumatic tetanic contraction of antagonist tendons of ossicular muscles. When the tendon of the tensor muscle of the tympanic membrane (fixing the neck of the malleus) and the stapedius tendon (attaching to the head of the stapes) simulteanously contract medial thrust of the incus and posterior pulling of the head of the stapes may concomitantly produce resulting in dislocation.

The reported case illustrates a typical case of incudostapedial disclocation. It is perfectly identified on classical transverse images and coronal oblique multiplanar reconstructions and the cardinal sign is the dissociation between the lenticular process of the incus and the head of the stapes. Our case also emphasizes the high diagnostic performances of 3D volume rendering of the ossicula.

## Competing Interests

The authors declare that they have no competing interests.
